# The Metropolitan Hospital

**Published:** 1893-05-06

**Authors:** 


### FESTIVAL DINNERS.

### THE METROPOLITAN HOSPITAL.

The annual dinner in aid of this hospital was held on Monday evening last at the Hôtel Metropole. The Duke of Portland presided, and amongst those present were the Lord Mayor, Lord Sandhurst, Sir Andrew Scoble, M.P., Sir James Paget, F.R.S., Mr. H. N. Custance, and the Secretary, Mr. C. Byers. Sir James Paget testified in his speech to the admirable manner in which the hospital was doing its work. The Lord Mayor also spoke of the importance of the charity. The Duke of Portland pointed out that the hospital being of modern construction had the advantage of the latest improvements in sanitation and in the arrangement of wards and offices. He remarked that unhappily the income of the hospital was decreasing. Modern specialism caused a tendency to develop special hospitals indefinitely, but general hospitals ought not to be allowed to suffer on this account. The deficit was over £5,000, and he hoped that the response to the present appeal would be worthy of themselves. Donations at the conclusion of the dinner were announced amounting to £2,318.

